# Supplementary material for: Operando monitoring of thermal runaway in commercial lithium-ion cells via advanced lab-on-fiber technologies
Source: Nat Commun. 2023 Aug 29;14:5251. doi: 10.1038/s41467-023-40995-3 (PMC10462619; doi:10.1038/s41467-023-40995-3)
Supplement: Supplementary file 1 — Supplementary Information [file 41467_2023_40995_MOESM1_ESM.pdf]

Supplementary Information

Operando monitoring of thermal runaway in commercial lithium-ion cells via advanced lab-on-fiber technologies

Wenxin Mei[a#], Zhi Liu[b#], Chengdong Wang[a], Chuang Wu[b], Yubin Liu[b], Pengjie Liu[a], Xudong Xia[b], Xiaobin Xue[b], Xile Han[b], Jinhua Sun[a], Gaozhi Xiao[c], Hwa-yaw Tam[d], Jacques Albert[e], Qingsong Wang[a]* and Tuan Guo[b]*

[a]*State Key Laboratory of Fire Science, University of Science and Technology of China, Hefei 230026, China*

[b]*Institute of Photonics Technology, Jinan University, Guangzhou 511443, China*

[c]*Advanced Electronics and Photonics Research Centre, National Research Council of Canada, Ottawa K1A 0R6, Canada*

[d]*Department of Electrical Engineering, The Hong Kong Polytechnic University, Kowloon, Hong Kong SAR, China*

[e]*Department of Electronics, Carleton University, Ottawa K1S 5B6, Canada*

[#]*These authors contributed equally to this work.*

**Correspondence: pinew@ustc.edu.cn (Q. Wang); tuanguo@jnu.edu.cn (T. Guo)*

The specifications of commercial 18650 lithium iron phosphate cell

Table S1 Specifications of commercial 18650 lithium iron phosphate cell.

Cell's parameter	Value
**Rated capacity**	1530 mAh @ 0.2 C discharge
**Size**	Diameter 18 mm, height 65 mm
**Weight**	40 ± 2.0 g
**Voltage range**	2.0 V ~ 3.65 V
**Internal resistance**	30 ~ 50 mΩ
**Rated voltage**	3.2 V @ 0.2 C discharge
**Cycle life**	Discharge capacity (0.5 C, 1000th cycle) > 80% SOH

The response spectra of FBG to temperature and pressure

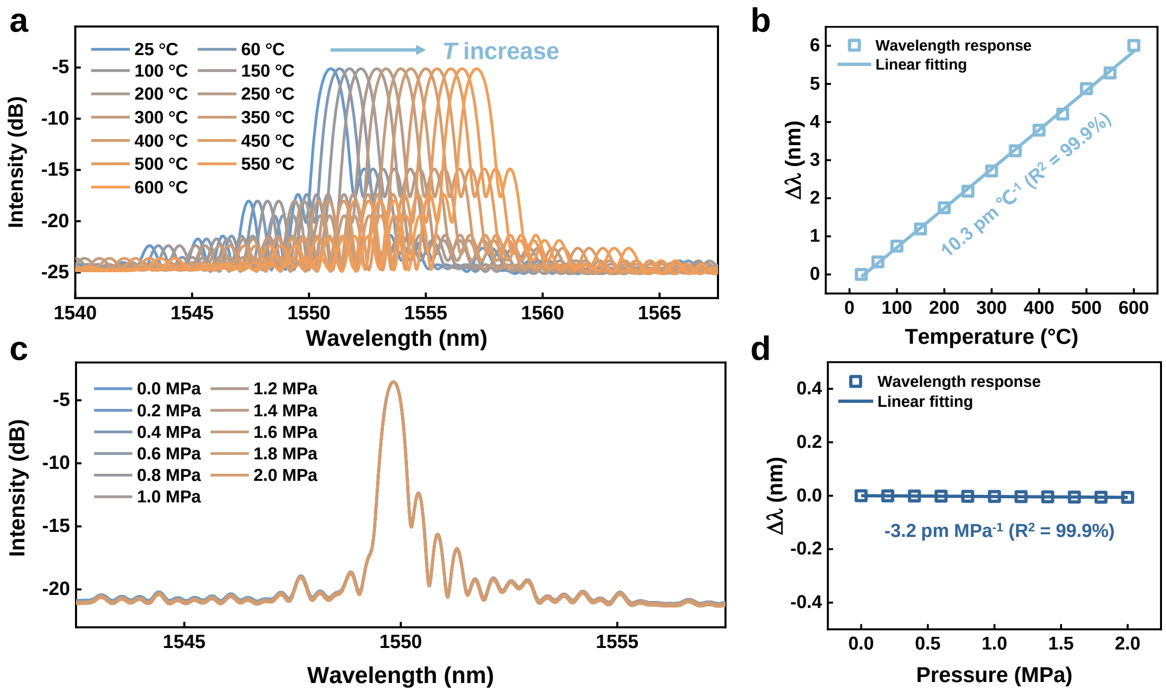

Fig. S1 Response spectra of FBG to temperature and pressure. (a) The full spectral response of FBG from 25 to 600°C, the arrows indicate the direction of wavelength change upon increase of temperature. (b) With the temperature sensitivity of 10.3 pm °C^{-1}. (c) The full spectral response of FBG sensor in air at 0–2 MPa in 0.2 MPa increment, there exhibits superior linear relationship (d) with the pressure sensitivity of -3.2 pm MPa^{-1}. (c and d) Wavelength response data (symbol) and linear fitting curve (solid line). Considering the pressure limit of the safety valve of the 18650 batteries, 2 MPa was selected here as the maximum pressure.

Response spectra of FPI to temperature and pressure

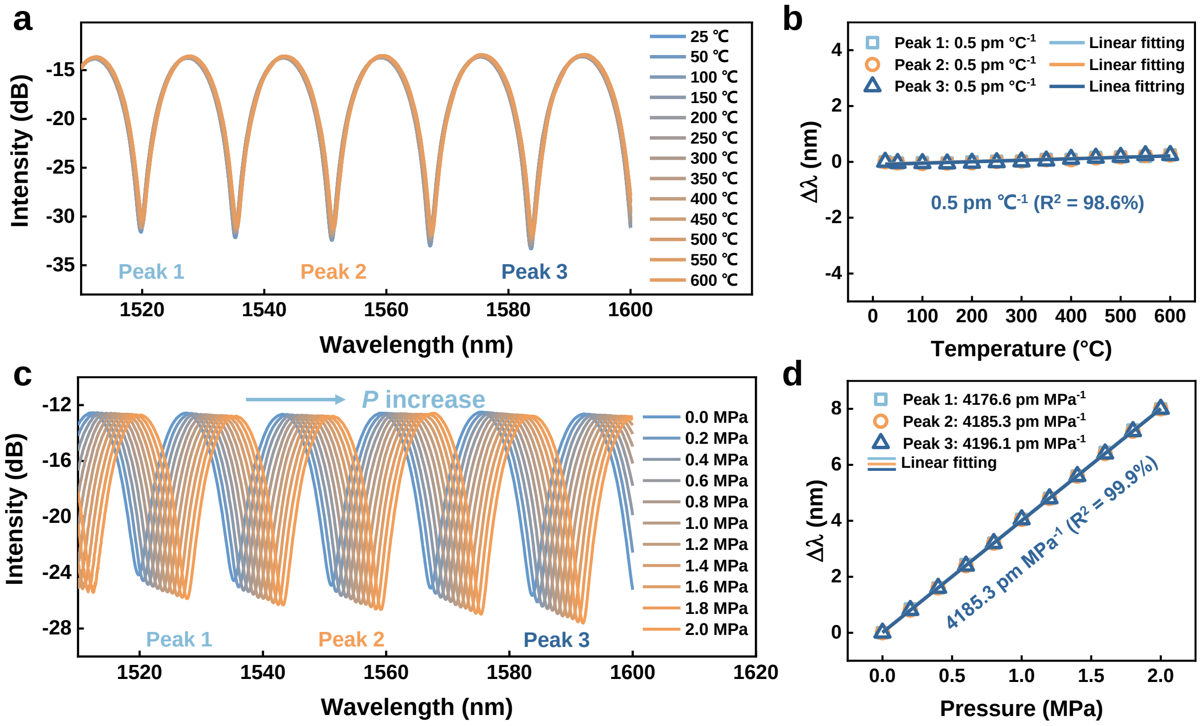

Fig. S2 Response spectra of FPI to temperature and pressure. (a) The full spectral response of FPI sensor from 25 to 600°C. The three resonance peaks of FPI at ~1520 nm, ~1550 nm and ~1580 nm as a function of *T*. (b and d) Raw data (symbol) and linear fitting curve (solid line). The temperature response sensitivity of FPI is 0.5 pm °C^{-1}. (c) The full spectral response of FPI sensor in air at 0–2 MPa in 0.2 MPa increment, the arrows indicate the direction of wavelength change upon increase of pressure. (d) The pressure response sensitivity of FPI is 4185.3 pm MPa^{-1}.

The temperature sensitivities of an FBG and an FPI are both determined by the following Eq. (S1)[1]:

$$\frac{\Delta\lambda}{\Delta T} = \left(\frac{\Delta n_{eff}}{n_{eff}} + \frac{\Delta L}{L}\right)\frac{\lambda}{\Delta \mathrm{T}} \approx (\delta + \alpha)\lambda \tag{S1}$$

where δ = 8.3×10^{-6} °C^{-1} and α = 0.55×10^{-6} °C^{-1} are the thermo-optic coefficient and linear thermal expansion coefficient of silica, respectively; λ is free space wavelength; $\Delta\lambda$ is the wavelength shift caused by external perturbations; ΔT is external temperature change; $\Delta L/L$ is strain. In practice, strain is eliminated by fixing the FBG-PPI assembly at only one end in a 0.5 mm diameter drilled central hole into the cell, where it remains suspended. For the air-cavity FPI, its temperature sensitivity is mainly attributed to the thermal expansion effect of the silica wall. Therefore, its temperature sensitivity is much lower than that of the FBG. In our experiment, the measured temperature sensitivities of the FPI and the FBG are 0.5 pm °C^{-1}

and 10.3 pm °C $^{-1}$, respectively. The "temperature insensitivity" is a relative concept. In many publications, they claim that their sensor is temperature insensitive when the sensitivity is less than 1 pm °C $^{-1}$. What's more, the pressure sensitivity of our FPI sensor is very high. The temperature-pressure cross-sensitivity coefficient is calculated to be (0.5 pm °C $^{-1}$)/(4185.3 pm MPa^{-1}) ≈ 1.2×10^{-4} MPa °C $^{-1}$. This means that a 100 °C temperature fluctuation only leads to a neglectable pressure measurement error of 0.01 MPa. So, we believe we could claim our FPI is "temperature insensitive".

Error assessment of the FBG temperature measurement

In order to achieve the error assessment of the FBG temperature measurement in both normal cycling conditions and thermal runaway conditions, the FBG sensor and thermocouple are both implanted into the 18650 cells simultaneously to character their temperature response. The positions of the FBG and TC are displayed in **Fig. S3a**. The Pearson correlation analysis[2,3] is used to quantify their relationship. The Pearson correlation coefficient (*PCC*) is calculated as[4]:

$$PCC = \frac{\sum_{i=1}^{n}\left(T_{FBG_i} - \overline{T_{FBG}}\right)\left(T_{TC_i} - \overline{T_{TC}}\right)}{\sqrt{\sum_{i=1}^{n}\left(T_{FBG_i} - \overline{T_{FBG}}\right)^2}\sqrt{\sum_{i=1}^{n}\left(T_{TC_i} - \overline{T_{TC}}\right)^2}} \qquad (S2)$$

where T_{FBG} and T_{TC} represent the temperature measured by FBG and TC, respectively. *PCC* value is between -1 and 1, where 1 or -1 represents a 100% linear relevance, and 0 means 0% relevance.

During a consecutive charge-discharge cycling at 0.5 C, 1 C, 1.5 C and 2 C with 30-minute relaxation setting between charge and discharge for temperature recovery, the internal temperature evolution of the FBG and TC is nearly identical as shown in **Fig. S3b**. The *PCC* is 99.86%, the maximum error between the temperature measured by FBG and TC during normal cycling is 0.12 °C, the relative error is merely 0.31%. And when the cell with 100% SOC is triggered thermal runaway by overheating of a 100 W heater, the internal temperature monitored by FBG and TC is also highly overlapped as shown in **Fig. S3c**. The *PCC* measured is 99.89%, the absolute error between the maximum temperature measured by FBG and TC during normal cycling is 2.43 °C, the relative error is merely 0.46% as shown in **Fig. S3d**.

Therefore, for both normal cycling conditions and thermal runaway conditions, the relative temperature errors between thermocouple and FBG are less than 0.5% and the *PCCs* are higher than 99.85%.

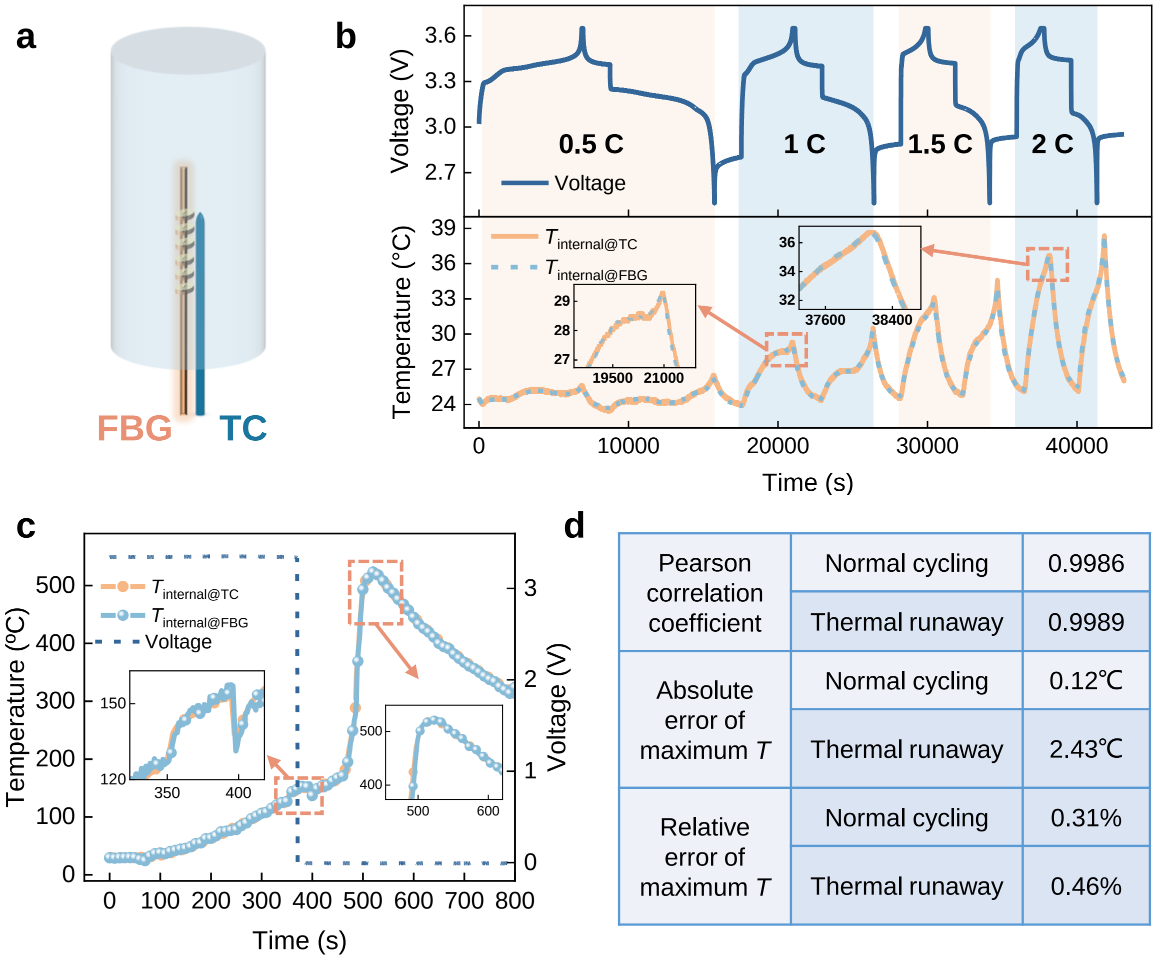

Pearson correlation coefficient	Normal cycling	0.9986
	Thermal runaway	0.9989
Absolute error of maximum *T*	Normal cycling	0.12℃
	Thermal runaway	2.43℃
Relative error of maximum *T*	Normal cycling	0.31%
	Thermal runaway	0.46%

Fig. S3 Error assessment of the FBG temperature measurement during charge-discharge cycling and thermal runaway. (a) The position of the implanted thermocouple and FBG. (b) The voltage and internal temperature evolution monitored by thermocouple and FBG during normal cycling at 0.5 C, 1 C, 1.5 C and 2 C, where a 30-minute relaxation is set between charge and discharge. (c) The voltage and internal temperature evolution monitored by thermocouple and FBG during thermal runaway of the cell with 100% SOC. (d) The Pearson correlation coefficient (*PCC*), absolute and relative error of maximum temperature during normal cycling and thermal runaway.

Pressure response spectra of FBG-FPI to different gas compositions

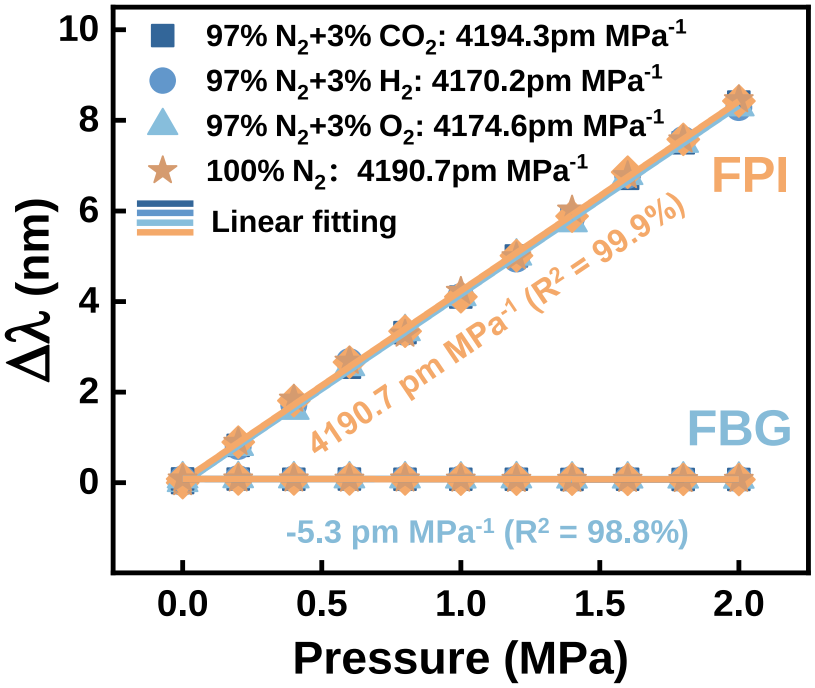

Fig. S4 The pressure response sensitivity of FBG-FPI, in which the FPI shows a linear pressure sensitivity (independent of different components of gases, i.e. 100% N_2 and 97% N_2 mixed with 3% CO_2/H_2/O_2), while the FBG shows insensitivity to pressure.

Implanting optical fiber sensor in cell and performing thermal runaway tests

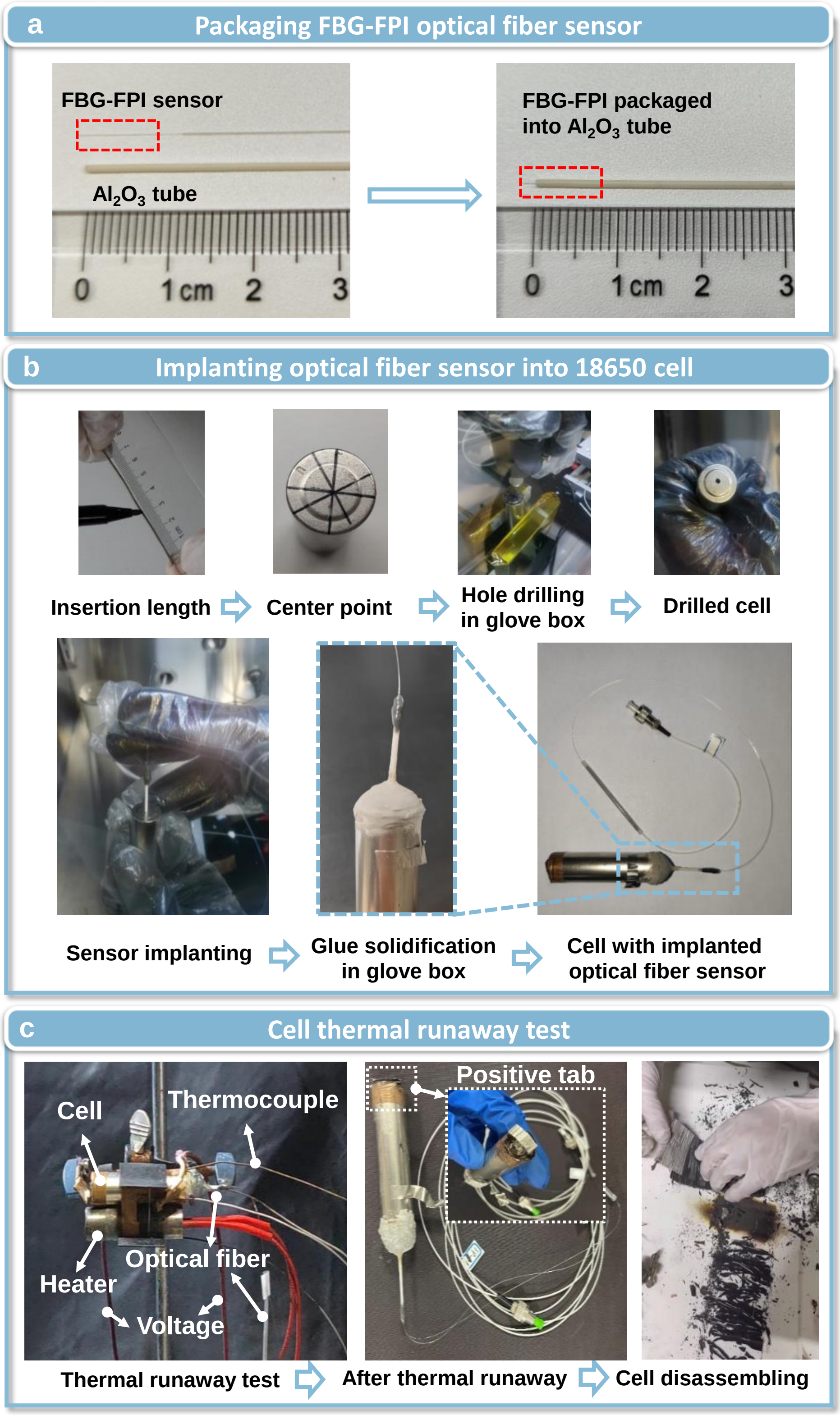

Fig. S5 All the processes of implanting optical fiber sensor in cell and performing thermal runaway tests: (a) tube-packaging FBG-FPI sensor, (b) implanting optical fiber sensor in cell and (c) cell thermal runaway tests.

Repeatability of FBG and FPI sensors before and after thermal runaway measurement

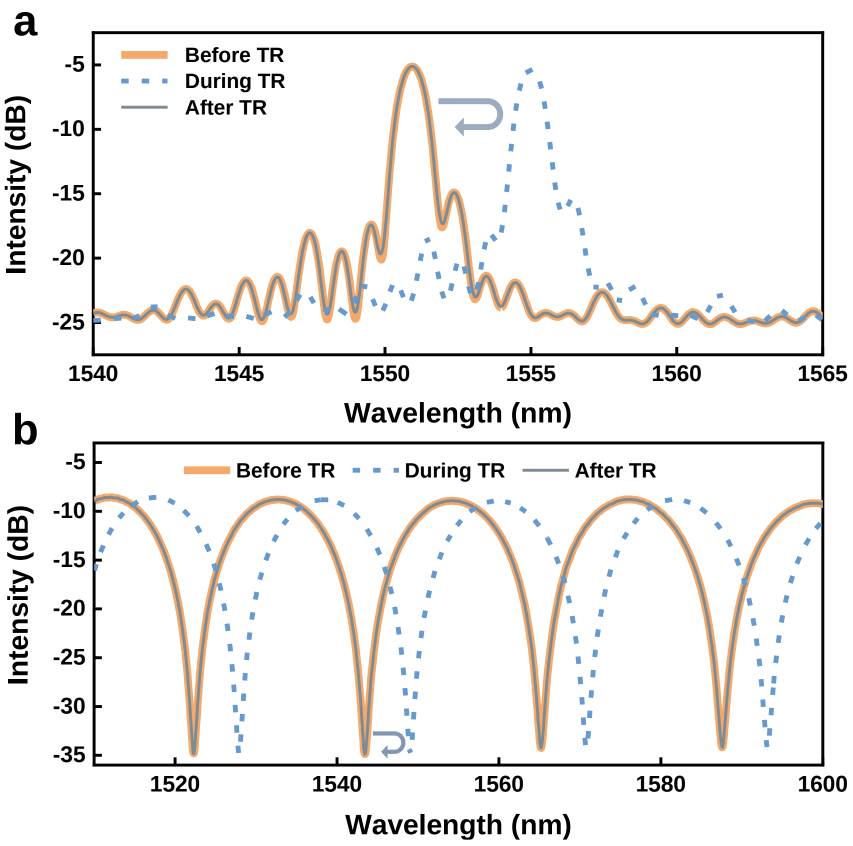

Fig. S6 The unaffected optical signal after thermal runaway. The spectrum of FBG (a) and FPI (b) before, during and after thermal runaway. “TR” is the abbreviation of thermal runaway.

Fabrication technologies of FPI optical fiber sensor

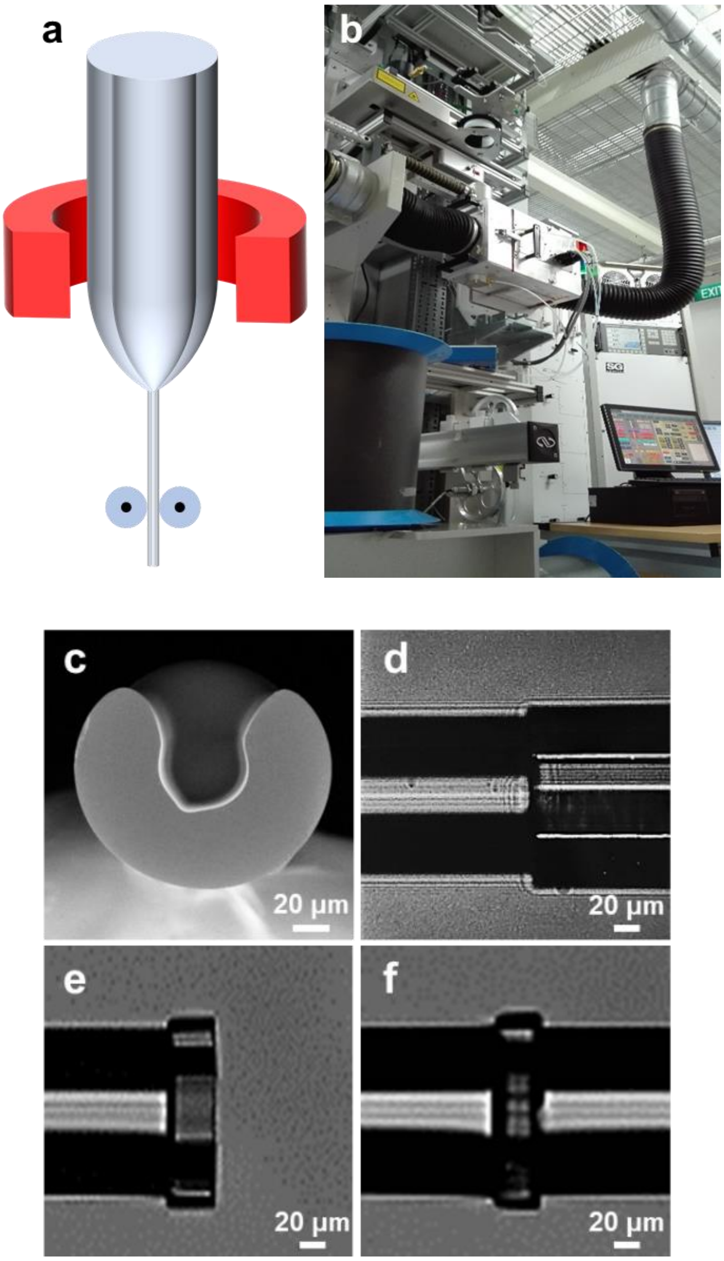

Fig. S7 Fabrication process of the FPI[5]. (a) Schematic diagram of the open-cavity fiber thermal drawing process. (b) Open cavity fiber draw tower. (c) SEM image of the cross-section of an open-cavity fiber. (d) Splicing a piece of open-cavity fiber to a SMF. (e) Cutting controlled length of open-cavity fiber. (f) Splicing the cut open-cavity fiber to another SMF.

The FPI's sensing characteristics with different cavity lengths

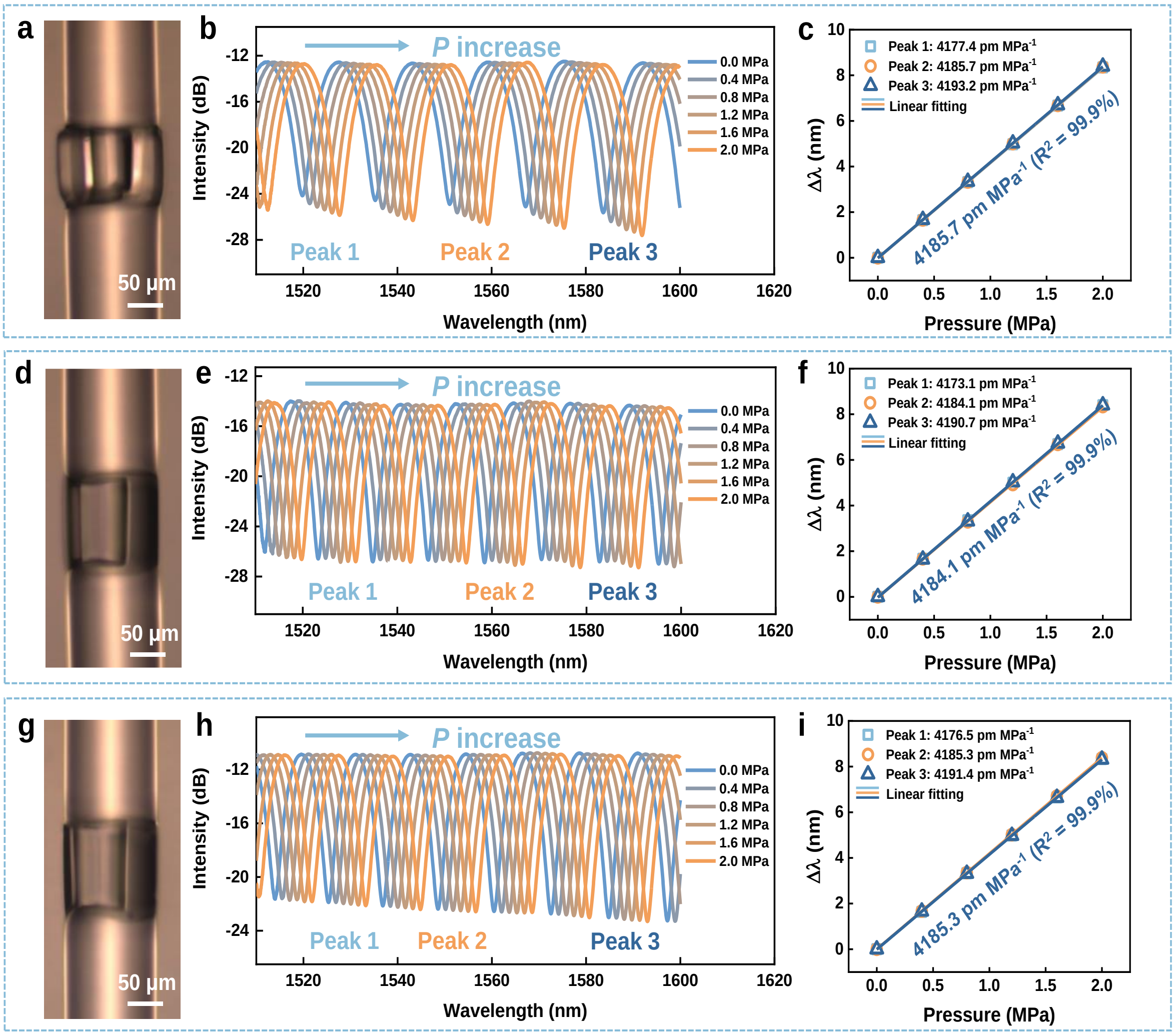

Fig. S8 Optical microscopy images and response curves to pressure for FPI with different cavity lengths. (a–c), (d–f), (g–i) indicate the response to pressure for FPI with cavity lengths L=65.86 μm, L=94.59 μm and L=92.97 μm, respectively. The arrows indicate the wavelengths of the three resonance peaks of FPI at ~1520 nm, ~1550 nm and ~1585 nm as a function of *P*. (c, f, i) Raw data (symbol) and linear fitted curve (solid line). (a, d, g) Optical microscope images of FPIs with different cavity lengths. (b, e, h) The full spectral response of FPI sensor, the arrows indicate the direction of wavelength change upon increase the pressure, the wavelength red shift with increasing *P*.

Fabrication technologies of FBG optical fiber sensor

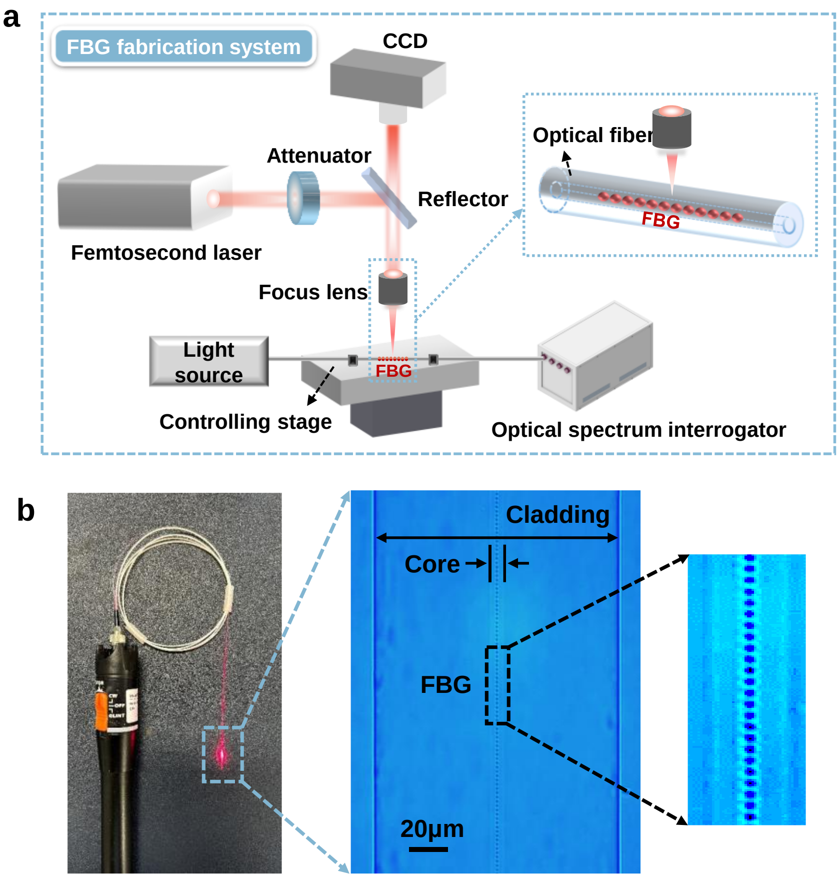

Fig. S9 Femtosecond-laser-inscribed FBG. (a) Femtosecond laser inscription system. (b) Photograph of FBG launched with red light and its zoomed microscope image.

Fabrication process of the FBG-FPI sensor

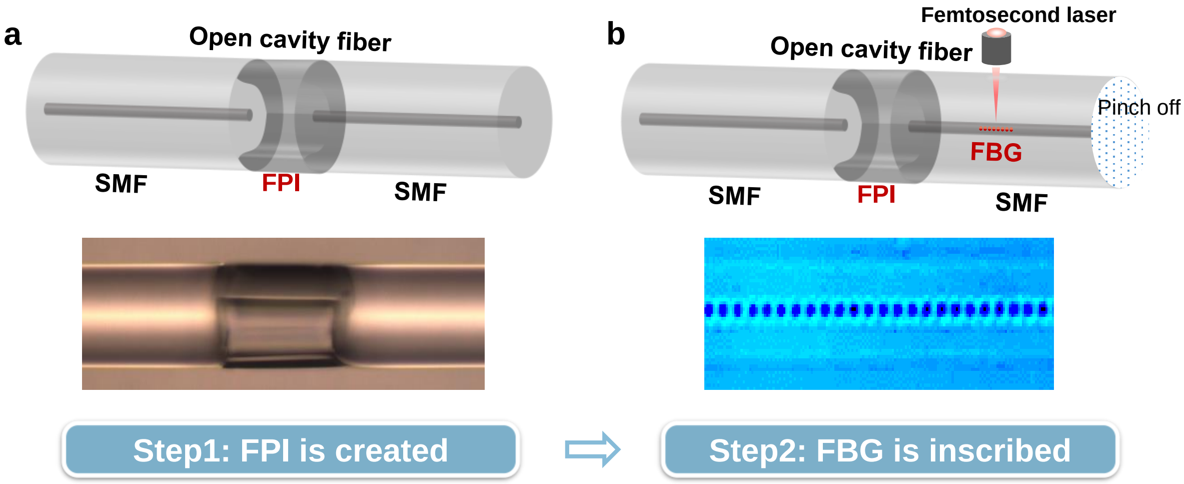

Fig. S10 Fabrication process of the FBG-FPI sensor. (a) The FPI is firstly created, and (b) the FBG is inscribed.

Heating mode and temperature

The thermal runaway is triggered by a heater attached to the cell, and the external heater is turned off near the beginning of Stage III since the internal processes drive the heat generation inside the cell at that time.

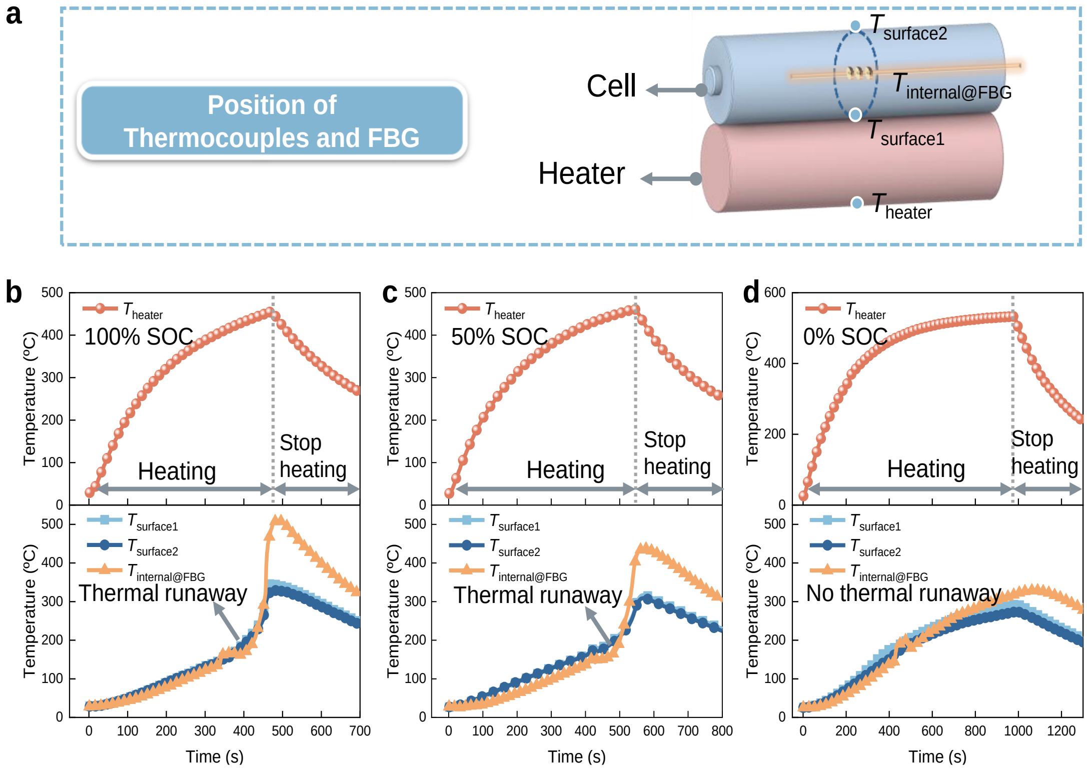

Fig. S11 The heating mode and temperature evolution of the cell with 100% SOC, 50% SOC and 0% SOC. (a) The position of thermocouples and FBG. (b,c,d) Temperature of heater and cell at three cases.

Summary of the characteristic parameters during thermal runaway

Table S2 Characteristic parameters of the cell with 0% SOC, 50% SOC and 100% SOC during thermal runaway

Characteristic parameters	Symbol (unit)		0% SOC	50% SOC	100% SOC
Characteristic temperature					
**Turning point**	T_{turn} (°C)	$T_{turn,s}$	58.7 (200 s)	83.3 (178s)	74.3 (162 s)
		$T_{turn,in}$	71.6	56.8	64.2
		$T_{turn,avg}$	65.2	70.0	69.2
**Voltage drop (internal short circuit)**	T_{ISC} (°C)	$T_{ISC,s}$	162.7 (426 s)	153.9 (421 s)	154.0 (353 s)
		$T_{ISC,in}$	154.3	172.8	163.0
**Venting**	T_{vent}(°C)	$T_{vent,s}$	161.5 (422 s)	171.7 (418 s)	157.2 (364 s)
		$T_{vent,in}$	148.4	152.1	167.1
**Trigger of thermal runaway**	T_{TR}(°C)	$T_{TR,s}$	/	215.4 (507 s)	194.1 (406 s)
		$T_{TR,in}$	/	220.3	171.4
**Maximum temperature rise rate**	$R_T(°C \cdot s^{-1})$	$R_{max,s}$	/	2.76 (542 s)	7.40 (455 s)
		$R_{max,in}$	/	8.84 (537 s)	35.23 (457 s)
**Maximum temperature**	T_{max}(°C)	$T_{max,s}$	273.3 (987 s)	308.8 (568 s)	328.8 (478 s)
		$T_{max,in}$	330.3 (1082 s)	438.1 (572 s)	509.8 (489 s)
**Maximum temperature difference**	ΔT_{max}(°C)		57	129.3	181.0
Characteristic pressure					
**First pressure peak**	P_1(MPa)		1.65	1.66	1.79
**Second pressure peak**	P_2(MPa)		/	0.38	0.58

Note: the subscripts "s" and "in" indicate "surface" and "internal", which represent the surface and internal temperature monitored by thermocouple and FBG, respectively. "/" indicates this parameter cam be ignored or does not exist.

Table S2 summarizes the characteristic parameters of the cell with 0% SOC, 50% SOC and 100% SOC during thermal runaway, it can be seen that the time and temperature at which safety venting occurs show little correlation with the SOC[6] as the electrolyte property is the same for different SOCs, which is attributed to that the safety venting relies heavily on the electrolyte evaporation but not on the generation of gases during chemical reactions[7]. With regards to the internal short circuit, it depends mostly of the separator melting and shrinkage and it displays weak correlations with SOC.

Characterizations of cell over thermal runaway using DSC, SEM, EDS, XRD methods

In-depth thermal runaway mechanism is revealed by post-mortem approaches involving DSC (Differential Scanning Calorimetry), SEM (Scanning Electron Microscope), EDS (Energy Dispersive Spectrometer) and XRD (X-rays Diffraction) in characterizing thermal stability, surface morphology, element composition and structure evolution experiencing thermal runaway, represented by the cell with 100% SOC.

- **DSC characterizations**

The heat flow of separator, the sample involving lithiated graphite anode, delithiated cathode and electrolyte are measured by the DSC 214 (NETZSCH) where the samples with electrolyte are encapsulated in high pressure gold-plated crucible with argon-filled glove box. All of the samples are performed with a ramp rate of 10 °C min^{-1}, where the separator is heated from ambient temperature to 200 °C and the sample of "cathode+anode+electrolyte" is heated from ambient temperature to 450 °C.

The heat flow of the separator (**Fig. S12a**) indicates that the separator melting starts at 158.0 °C and is followed by the further shrinkage at about 164.0 °C, and the collapse ends at 171.0 °C, which explains exactly that the internal short circuit temperature at the cell with different SOCs all locates within this range as summarized in **Table S2**. The first exothermic peak of heat flow of the combination of fully lithiated graphite, fully delithiated LFP and electrolyte (**Fig. S12b**) is associated with SEI decomposition[8] appearing from 84.4 °C, which is the beginning of parasitic side reaction to generate gases for further internal pressure accumulation. Subsequently, the second exothermic peak characterizes the reactions between active materials and electrolyte with the massive reactions starting at 168.4 °C and peaking at 242.4 °C, which is correlated to the trigger temperature of thermal runaway ($T_{\mathrm{TR,s}}$=194.1 °C and $T_{\mathrm{TR,s}}$=171.4 °C), implying that the reactions between active materials and electrolyte occupy the dominant position in triggering thermal runaway. Finally, the third slight

exothermic peak represents the reaction between graphite electrode with binder[9] from 289.4 °C, resulting in sustained gases generation and accelerated temperature rise rate[10].

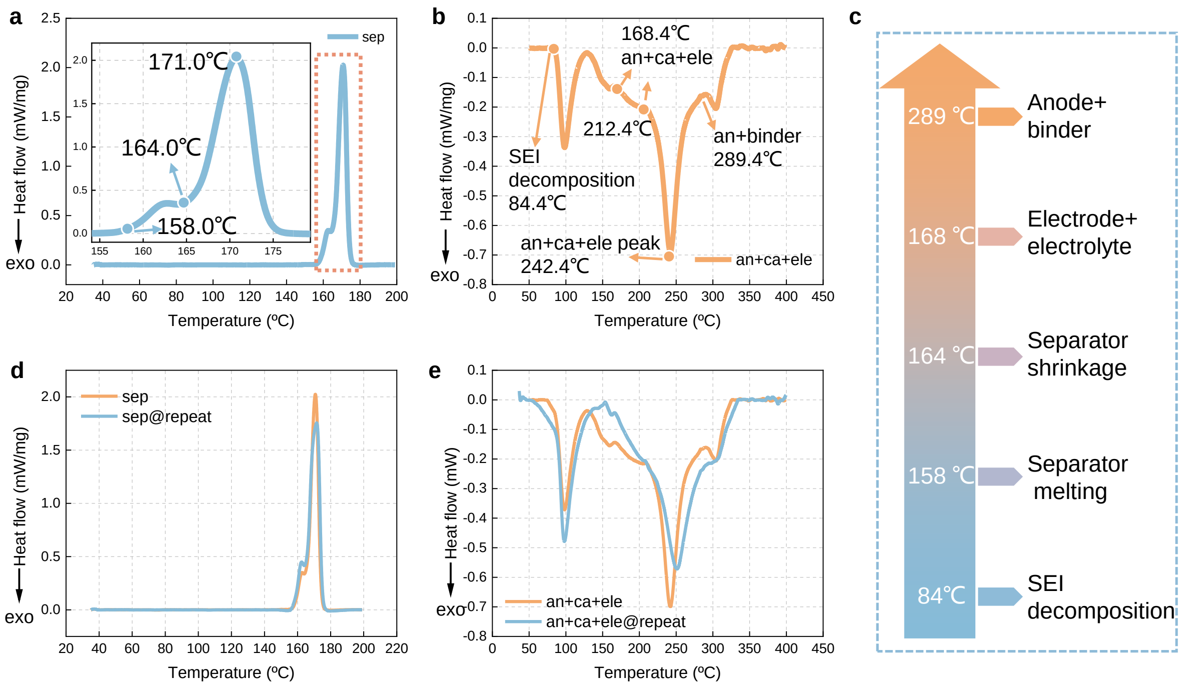

Fig. S12 The DSC results and the derived side reaction sequence to reveal thermal runaway mechanism. (a, b) The heat flow curves of separator (a) and graphite in fully lithiated state, LFP in fully delithiated state with electrolyte (b) monitored by DSC. (c) The side reaction sequence with the corresponding temperature. (d, e) The repeated DSC curves of separator (d) and an+ca+ele (e).

- **SEM-EDS characterizations**

The SEM-EDS patterns of cathode and anode before/after thermal runaway are acquired by Gemini SEM 500 manufactured by ZEISS. The as-prepared samples before thermal runaway are pasted on a self-made container by conductive glue in glove box, and then quickly put on the SEM-EDS equipment within 30-min after taking container out of the glove box. The as-prepared samples after thermal runaway are directly pasted on the sample table in the air. SEM images, EDS images and EDS spectrum are all obtained.

SEM patterns of anode before thermal runaway with 100%SOC are smooth and flat with the distinct lithiated graphite particles (**Fig. S13a** and **b**). Yet there seems to be plenty of impurities covering the graphite surface to lead to a rough pattern showing in **Fig. S13e** and **f**, which is estimated to be decomposition products after thermal runaway. The EDS mapping and spectrum in **Fig. S13c**, **d**, **g**, **h** further confirm this conjecture, where the additional fluorine

(F) element with intensified signal is detected on the anode surface after thermal runaway, demonstrating that F-containing substances decomposed from electrolyte are accumulated on anode surface after thermal runaway. It is not coincidence but appeared at repeated samples provided in **Fig. S14**. The analogous rough SEM images are also discovered at cathode after thermal runaway as shown in **Fig. S13i**, **j**, **m**, **n**, while difference emerges at merely sporadic impurities appearing on cathode surface rather than covering the whole cathode surface like anode. Although the EDS patterns imply no variation in element distribution and composition for cathode. Remarkably, compared with the SEM images of cathode before thermal runaway, the non-uniform particle size distribution together with indistinct particle boundaries are discovered after thermal runaway, indicating the changed structure that will be disclosed by XRD displayed in **Fig. S15.**

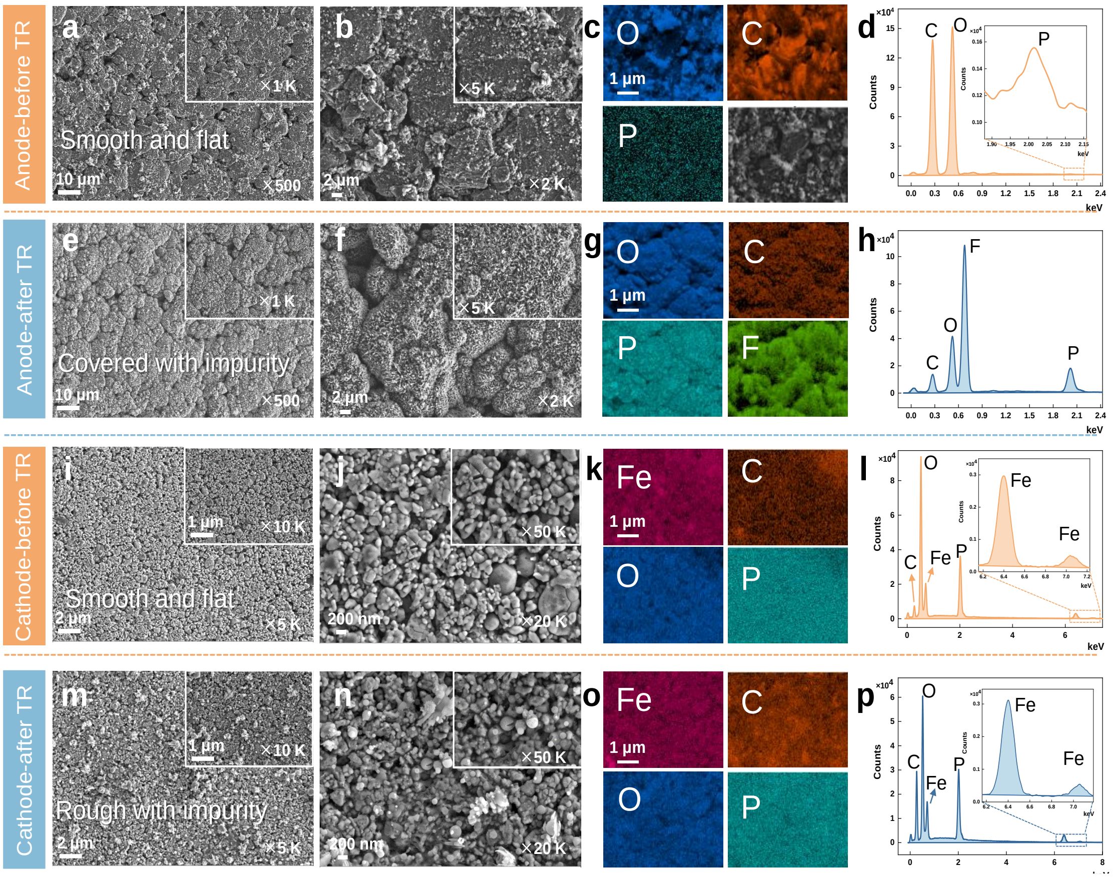

Fig. S13 The SEM-EDS patterns exhibiting surface morphology and elemental composition before and after thermal runaway. (a–d) Anode before thermal runaway. (e–h) Anode after thermal runaway. (i–l) Cathode before thermal runaway. (m–p) Cathode after thermal runaway. a, b, e, f, I, j, m, n, SEM pattern. c, g, k, o, EDS mapping. d, h, l, p, EDS spectrum.

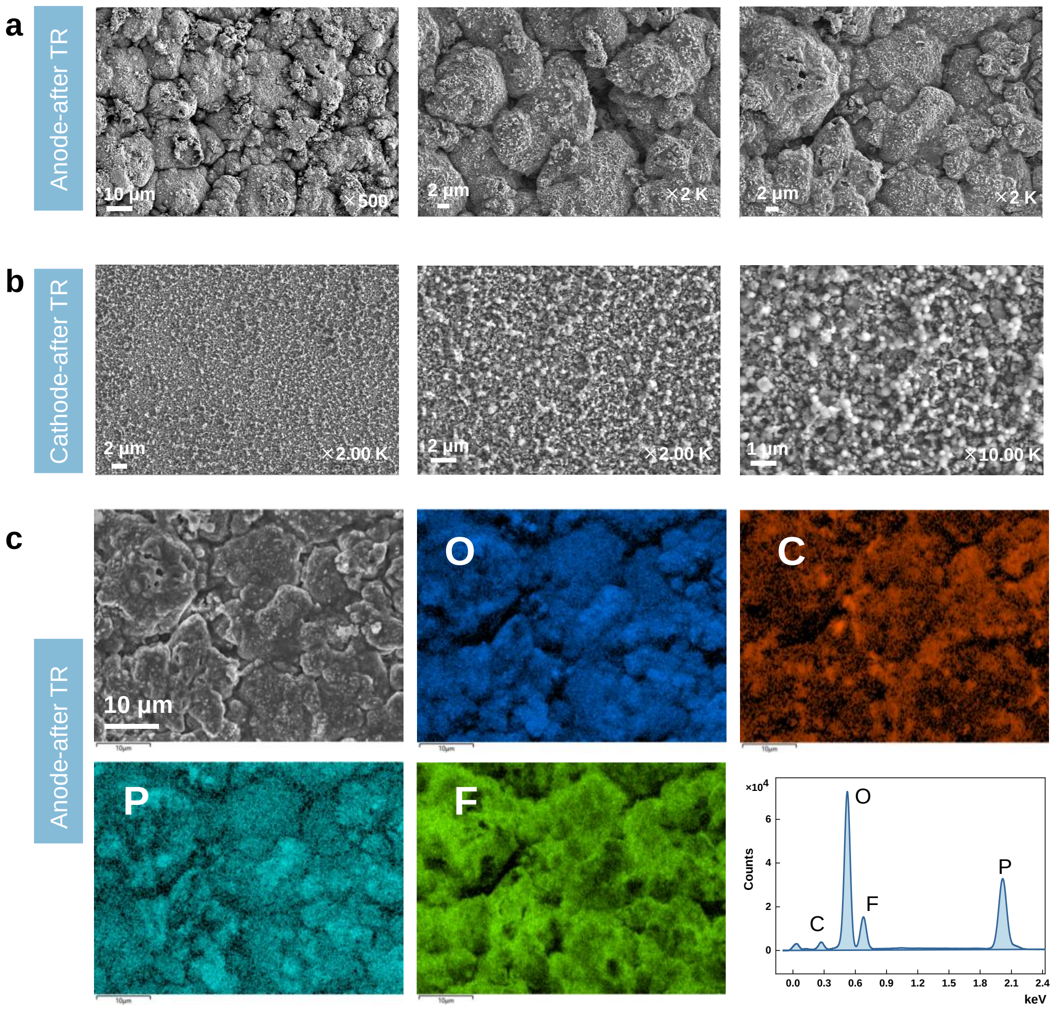

Fig. S14 The repeated SEM-EDS patterns demonstrating rough surface morphology with impurities and additional fluorine (F) element after thermal runaway. (a) The anode surface morphology after thermal runaway. (b) The cathode surface morphology after thermal runaway. (c) The EDS mapping and spectrum of the anode after thermal runaway.

- **XRD characterizations**

The XRD pattern of cathode and anode before/after thermal runaway are acquired by Smartlab (Cu Kα) manufactured by Rigaku to reveal the structure evolution during thermal runaway. The anode materials before thermal runaway are scraped off from current collector and ground into powder in glove box, while anode after thermal runaway and cathode are operated with the same procedures in the air.

In **Fig. S15a** all diffraction peaks before thermal runaway manifest excellent consistency with the standard card of the delithiated LFP, while divergence appears at cathode after thermal runaway that the predominant crystalline phase detected by XRD is $Fe_2P_2O_7$ and

$Fe_7(PO_4)_6$, demonstrating that LFP has been decomposed with the existence of electrolyte[11,12]. This observation matches well with the predicted three-step decomposition of $FePO_4$ using DFT calculations [13] as expressed in Eq.(1).

$$FePO_4 \rightarrow 0.1(Fe_7(PO_4)_6 + Fe_3(P_2O_7)_2) \rightarrow 0.167(Fe_3(PO_4)_2 + Fe_3(P_2O_7)_2) \rightarrow 0.5Fe_2P_2O_7 (1)$$

As for anode, the existed XRD peaks of LiC_6 and LiC_{12} provide evidence for the lithiated graphite[14] before thermal runaway, the coexistence peak of graphite-2H and graphite-3R is attributed to the unutilized graphite ascribed as the excessive anode capacity in design to avoid Li plating. Similarly, in **Fig. S15b** and **c** XRD pattern of anode after thermal runaway exhibits the highly typical graphite crystallized structure with the sharp coexistence peak of graphite-2H and graphite-3R, implying that the intercalated Li can be removed from lithiated graphite at elevated temperature to reserve the graphite structure. This can be rationalized by considering that Lewis acid PF_5 decomposed by $LiPF_6$ facilitates Li diffusion by removing electrons from graphite[15,16].

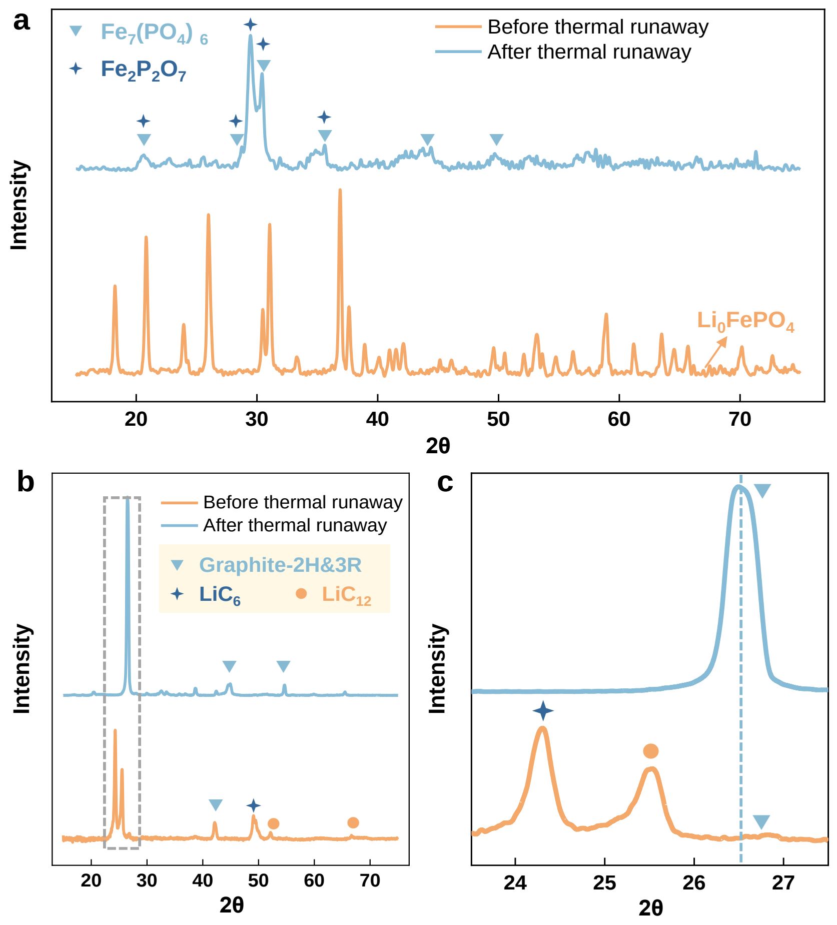

Fig. S15 The XRD evidences of cathode and anode before/after thermal runaway revealing structure evolution. (a) XRD patterns of cathode. (b) XRD patterns of anode and zoomed section (c).

FBG-FPI spectral interrogation system and compact power detection system

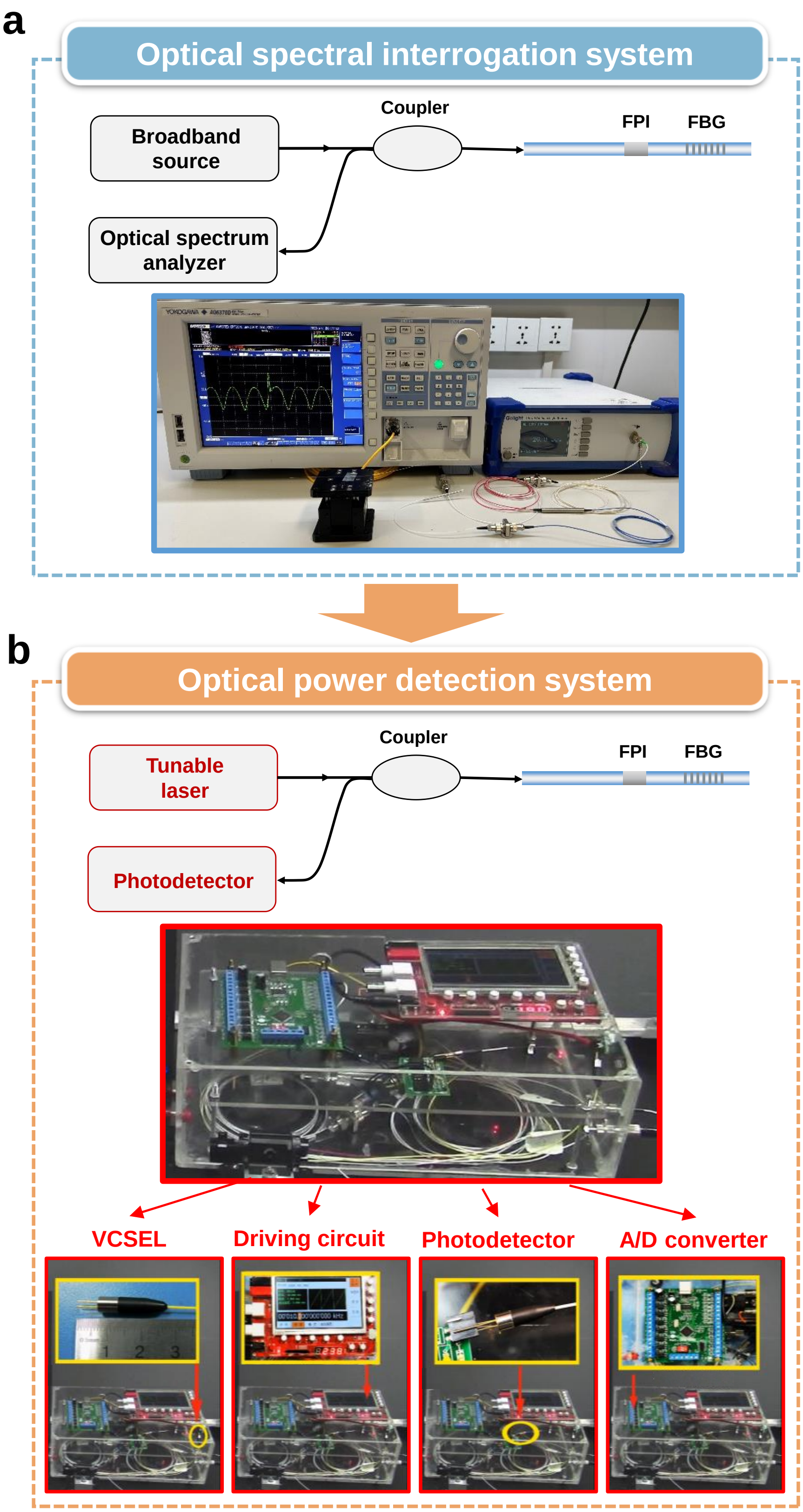

Fig. S16 The FBG-FPI spectral interrogation system and compact power detection system. (a) Present lab-based spectral interrogation system and (b) portable power detection instrumentation for in-field measurement.

Two different optical interrogation systems for sensor fabrication and cell monitoring

Table S3 Detailed setup parameters for different interrogation system.

Instrumentations	Experimental labs	Parameters
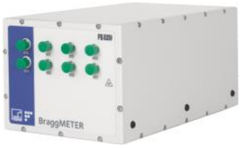 **FS22SI, HBM**	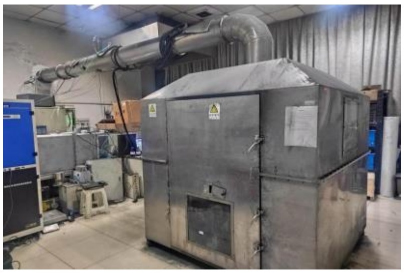 State Key Laboratory of Fire Science, University of Science and Technology of China	• Channel: 8<br>• Measurement time: 1 s<br>• Range: 1500-1600 nm<br>• Resolution: 1 pm<br>• 100001 points per samples
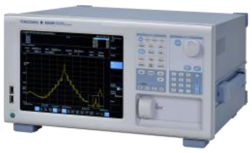 **AQ6380, YOKOGAWA**	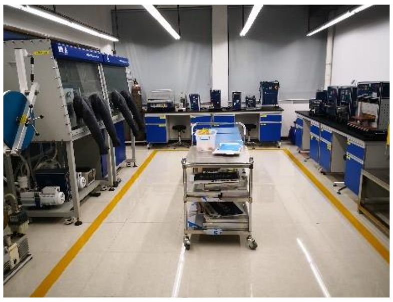 Institute of Photonics Technology, Jinan University	• Channel: 1<br>• Measurement time: 16 s<br>• Range: 1500-1600 nm<br>• Resolution: 1 pm<br>• 100001 points per samples (HIGH1 mode)

References

1. Wu, C., Fu, H. Y., Qureshi, K. K., Guan, B. O. & Tam, H. Y. High-pressure and high-temperature characteristics of a Fabry-Perot interferometer based on photonic crystal fiber. *Opt. Lett.* **36**, 412-414 (2011).
2. Zhou, Y., Huang, M., Chen, Y. & Tao, Y. A novel health indicator for on-line lithium-ion batteries remaining useful life prediction. *J. Power Sources* **321**, 1-10 (2016).
3. Ma, Y., Shan, C., Gao, J. & Chen, H. A novel method for state of health estimation of lithium-ion batteries based on improved LSTM and health indicators extraction. *Energy* **251**, 123973 (2022).
4. Conover, W. J. *Practical nonparametric statistics*. Vol. 350 (john wiley & sons, 1999).
5. Wu, C., Liu, Z., Zhang, A. P., Guan, B.-O. & Tam, H.-Y. In-line open-cavity Fabry-Perot interferometer formed by C-shaped fiber fortemperature-insensitive refractive index sensing. *Opt. Express* **22**, 21757-21766 (2014).
6. Zhong, G., Mao, B., Wang, C., Jiang, L., Xu, K., Sun, J. & Wang, Q. Thermal runaway and fire behavior investigation of lithium ion batteries using modified cone calorimeter. *J. Therm. Anal. Calorim.* **135**, 2879-2889 (2019).
7. Qin, P., Sun, J. & Wang, Q. A new method to explore thermal and venting behavior of lithium-ion battery thermal runaway. *J. Power Sources* **486**, 229357 (2021).
8. Zu, C. X., Yu, H. G. & Li, H. Enabling the thermal stability of solid electrolyte interphase in Li-ion battery. *Infomat* **3**, 648-661 (2021).
9. Du Pasquier, A., Disma, F., Bowmer, T., Gozdz, A. S., Amatucci, G. & Tarascon, J. M. Differential scanning calorimetry study of the reactivity of carbon anodes in plastic Li-ion batteries. *J. Electrochem. Soc.* **145**, 472-477 (1998).
10. Jin, Y., Zheng, Z., Wei, D., Jiang, X., Lu, H., Sun, L., Tao, F., Guo, D., Liu, Y., Gao, J. & Cui, Y. Detection of Micro-Scale Li Dendrite via H-2 Gas Capture for Early Safety Warning. *Joule* **4**, 1714-1729 (2020).
11. Röder, P., Baba, N., Friedrich, K. A. & Wiemhöfer, H. D. Impact of delithiated Li0FePO4 on the decomposition of LiPF6-based electrolyte studied by accelerating rate calorimetry. *J. Power Sources* **236**, 151-157 (2013).
12. Kim, J., Park, K.-Y., Park, I., Yoo, J.-K., Hong, J. & Kang, K. Thermal stability of Fe–Mn binary olivine cathodes for Li rechargeable batteries. *J. Mater. Chem.* **22**, 11964-11970 (2012).
13. Ong, S. P., Jain, A., Hautier, G., Kang, B. & Ceder, G. Thermal stabilities of delithiated olivine MPO4 (M=Fe, Mn) cathodes investigated using first principles calculations. *Electrochem. Commun.* **12**, 427-430 (2010).
14. Reynier, Y., Yazami, R. & Fultz, B. XRD evidence of macroscopic composition inhomogeneities in the graphite–lithium electrode. *J. Power Sources* **165**, 616-619 (2007).
15. Yang, H., Bang, H., Amine, K. & Prakash, J. Investigations of the Exothermic Reactions of Natural Graphite Anode for Li-Ion Batteries during Thermal Runaway. *J. Electrochem. Soc.* **152**, A73 (2005).
16. Lee, H. H., Wan, C. C. & Wang, Y. Y. Thermal Stability of the Solid Electrolyte Interface on Carbon Electrodes of Lithium Batteries. *J. Electrochem. Soc.* **151**, A542-A547 (2004).
