# Supplementary material for: Operando monitoring of thermal runaway in commercial lithium-ion cells via advanced lab-on-fiber technologies
Source: Nat Commun. 2023 Aug 29;14:5251. doi: 10.1038/s41467-023-40995-3 (PMC10462619; doi:10.1038/s41467-023-40995-3)
Supplement: Supplementary file 3 — Description of Additional Supplementary Information [file 41467_2023_40995_MOESM3_ESM.pdf]

Description of additional supplementary files

Supplementary Movie 1 : The thermal runaway process of the cell with 50%SOC.
